# Advances in the application of gene methylation detection in cervical cancer screening

**DOI:** 10.3389/fonc.2026.1920299

**Published:** 2026-09-24

**Authors:** Han Xiao, Fei Wang, Yanping Wang, Yuqin Liu, Liehong Wang

**Affiliations:** 1 College of Clinical Medicine, Qinghai University, Xining, Qinghai, China; 2 Internal Medicine of Traditional Chinese Medicine, Xining Yongchang Integrated Traditional Chinese and Western Medicine Clinic, Xining, Qinghai, China; 3 Department of Gynaecology and Obstetrics, Qinghai Red Cross Hospital, Xining, Qinghai, China

**Keywords:** biomarkers, cervical cancer, epigenetics, gene methylation, HPV, screening

## Abstract

Cervical cancer remains a leading malignancy among women globally, with its pathogenesis strongly associated with persistent high-risk human papillomavirus (HPV) infection. Despite the widespread adoption of HPV testing in cervical cancer screening, its suboptimal specificity frequently results in overdiagnosis and overtreatment. Gene methylation, a key epigenetic modification, has emerged as a highly specific biomarker associated with cervical precancerous lesions and carcinogenesis, holding significant promise for enhancing screening precision. This review comprehensively summarizes recent advances in gene methylation detection for cervical cancer screening, with a focus on the identification of methylation biomarkers, the evolution of detection technologies, and their clinical utility. Furthermore, it addresses the current challenges and limitations in implementing methylation-based assays in routine clinical practice. By offering a systematic overview of the field, this work aims to support the optimization of cervical cancer screening strategies and promote the integration of epigenetic biomarkers into standard clinical workflows.

## 1 Introduction

Cervical cancer remains a significant global health challenge, particularly in low- and middle-income countries, where it ranks as one of the leading causes of cancer-related morbidity and mortality among women (1). The primary etiological factor for cervical carcinogenesis is persistent infection with high-risk human papillomavirus (hrHPV), which initiates a cascade of molecular and cellular events culminating in the development of cervical intraepithelial neoplasia (CIN) and invasive cervical cancer. Effective screening programs are critical for early detection and prevention, substantially reducing both incidence and mortality rates. Traditional cervical cancer screening has relied heavily on cytology-based methods, such as the Papanicolaou (Pap) smear, which, despite its historical success, suffers from limitations including variable sensitivity and the need for repeated testing (2). The advent of hrHPV DNA testing has improved sensitivity for detecting high-grade lesions but is hampered by suboptimal specificity, leading to increased colposcopy referrals and potential overtreatment (3). Consequently, there is an urgent need for more precise triage tools that can accurately stratify hrHPV-positive women according to their risk of progression to high-grade lesions or cancer.

In this context, aberrant DNA methylation of host genes has emerged as a promising biomarker for cervical cancer screening and triage. DNA methylation, an epigenetic modification involving the addition of methyl groups to cytosine residues in CpG dinucleotides, plays a pivotal role in regulating gene expression and maintaining genomic stability. Dysregulation of DNA methylation patterns is a hallmark of carcinogenesis, including cervical cancer, where hypermethylation of **host** tumor suppressor gene promoters and global hypomethylation contribute to malignant transformation. (Here, we refer exclusively to host cellular genes, not viral DNA.) Importantly, aberrant methylation changes can be detected early in the disease process, often preceding morphological alterations, making them attractive candidates for early diagnosis and risk stratification (4). Moreover, methylation markers have demonstrated potential not only in triaging hrHPV-positive women but also in predicting lesion progression and treatment response, thereby enhancing personalized management strategies.

Recent advances in molecular technologies have facilitated the identification and validation of numerous methylation markers associated with cervical neoplasia. For instance, genes such as PAX1, SOX1, ZNF671, EPB41L3, JAM3, and FAM19A4 have been extensively studied and shown to exhibit increased methylation that correlates with lesion severity. Panels combining multiple methylation markers have further improved diagnostic accuracy, achieving sensitivities and specificities that compare favorably with or surpass conventional cytology and HPV genotyping. Additionally, methylation assays have been successfully applied to various sample types, including clinician-collected cervical scrapes, self-collected vaginal samples, and even urine, broadening their applicability and potential to increase screening coverage, especially among underscreened populations. The integration of methylation testing into screening algorithms has shown promise in reducing unnecessary colposcopy referrals while maintaining high detection rates for CIN2+ and CIN3+ lesions.

The biological underpinnings of DNA methylation alterations in cervical carcinogenesis are closely linked to hrHPV infection and viral oncogene activity. The HPV E7 oncoprotein, for example, interacts with host epigenetic regulators such as DNA methyltransferases (DNMTs) and histone deacetylases, leading to epigenetic silencing of tumor suppressor genes including CDH1 and others involved in cell adhesion and immune response. Furthermore, methylation changes are associated with viral integration and host genome instability, contributing to lesion progression. These mechanistic insights reinforce the rationale for targeting DNA methylation as a biomarker for early detection and risk assessment.

Despite these advances, challenges remain in translating methylation biomarkers into routine clinical practice. Variability in marker panels, assay platforms, and cutoff thresholds necessitates further standardization and validation across diverse populations and clinical settings. Moreover, the psychological impact of screening and triage results on patients underscores the need for clear communication and counseling strategies (5). Ongoing research is also exploring the integration of methylation testing with other molecular markers and artificial intelligence to enhance diagnostic precision and cost-effectiveness.

In summary, gene methylation detection represents a rapidly evolving and highly promising approach to improve cervical cancer screening and triage. By enabling more accurate identification of women at risk for high-grade lesions and cancer, methylation biomarkers have the potential to optimize screening efficiency, reduce unnecessary interventions, and ultimately contribute to the global effort to eliminate cervical cancer as a public health problem. This review aims to systematically summarize the current research landscape on gene methylation detection in cervical cancer screening, focusing on marker selection, detection methodologies, clinical performance, and future directions for clinical application.

## 2 Current status and challenges of cervical cancer screening

### 2.1 Limitations of traditional screening methods

Traditional cervical cancer screening methods, including cytology (Pap smear) and human papillomavirus (HPV) testing, have been the cornerstone of cervical cancer prevention programs worldwide. However, these methods exhibit inherent limitations that affect their overall effectiveness and clinical utility. Cytology, which relies heavily on the morphological assessment of cervical cells by pathologists, suffers from moderate sensitivity, generally ranging from approximately 50% to 70%. This sensitivity is particularly inadequate for detecting adenocarcinomas, a subtype of cervical cancer that arises from glandular cells and is often less accessible to cytological sampling. The dependence on the subjective expertise of pathologists introduces variability and potential for missed diagnoses, especially in low-resource settings or where cytological expertise is limited (2).

HPV testing, on the other hand, offers superior sensitivity exceeding 90% for detecting high-risk HPV infections associated with cervical neoplasia. Despite this high sensitivity, HPV testing is plagued by low specificity. This is largely due to the high prevalence of transient HPV infections, especially in younger women under 30 years of age, which often resolve spontaneously without progressing to neoplasia. Consequently, a significant proportion of HPV-positive results represent transient infections rather than clinically significant lesions, leading to a high rate of false positives. This low specificity results in unnecessary referrals for colposcopy and potential overtreatment, imposing psychological distress on patients and increasing healthcare costs (6).

To mitigate these limitations, combined screening strategies integrating cytology and HPV testing have been adopted. While combined screening improves detection rates of cervical intraepithelial neoplasia (CIN) and invasive cancer, it also escalates the financial burden on healthcare systems and amplifies the psychological impact on patients due to increased follow-up procedures. Moreover, the management of HPV-positive young women remains controversial, as the natural history of HPV infections in this demographic often involves spontaneous clearance, raising concerns about overtreatment and its associated morbidity (6).

The limitations of traditional screening methods underscore the need for more precise triage tools that can accurately distinguish between transient HPV infections and lesions with malignant potential. For example, methylation of tumor suppressor genes such as PAX1 and SOX1 has demonstrated higher specificity in detecting high-grade lesions compared to HPV testing alone. Additionally, methylation panels such as FAM19A4/miR124-2 have shown consistent performance across diverse populations and sample types, suggesting their potential utility as triage tests to reduce unnecessary colposcopy referrals.

In summary, while cytology and HPV testing remain foundational in cervical cancer screening, their limitations—moderate sensitivity of cytology, low specificity of HPV testing, and the complexities introduced by combined screening—highlight the necessity for novel, more accurate, and cost-effective triage methods. Integration of molecular assays, particularly DNA methylation-based tests, may address these challenges by improving risk stratification, reducing overtreatment, and ultimately enhancing the effectiveness of cervical cancer screening programs.

### 2.2 Advantages of epigenetic biomarkers

Epigenetic biomarkers, particularly DNA methylation alterations, offer significant advantages in the early detection and risk stratification of cervical intraepithelial neoplasia grade 2 or worse (CIN2+), which are precancerous lesions with a high potential to progress to cervical cancer. One of the foremost advantages is the ability to detect DNA methylation changes at the precancerous stage, well before invasive cancer develops. These methylation alterations are not only detectable in CIN2+ lesions but also show a strong correlation with the risk of lesion progression, thereby serving as predictive markers for disease advancement. This early detection capability is crucial for timely intervention and improved patient outcomes. Furthermore, DNA methylation markers exhibit tissue specificity, enabling them to distinguish among transient human papillomavirus (HPV) infections, low-grade lesions, and high-grade lesions (4, 9). This specificity is instrumental in reducing false-positive results that often arise in HPV DNA testing, thereby enhancing the accuracy of cervical cancer screening programs. For instance, methylation of host genes such as FAM19A4 and miR124-2 has demonstrated high specificity in identifying clinically relevant cervical lesions, improving triage strategies for HPV-positive women. Another practical advantage of methylation biomarkers lies in their versatility regarding sample sources. DNA methylation can be reliably detected in cervical exfoliated cells collected during routine screening, self-collected vaginal samples, and even in liquid biopsies such as circulating tumor DNA (ctDNA) from blood or urine. This flexibility facilitates large-scale screening initiatives, especially in resource-limited settings where access to clinical facilities and trained personnel may be constrained. Self-sampling approaches combined with methylation testing have shown promise in increasing screening coverage and compliance, particularly among underscreened populations. Moreover, the stability of DNA methylation marks in various sample types supports their use in non-invasive or minimally invasive screening modalities. Collectively, these advantages underscore the potential of DNA methylation-based epigenetic biomarkers to enhance cervical cancer screening by enabling early, accurate, and accessible detection of precancerous lesions, thereby optimizing patient management and reducing the burden of cervical cancer globally (7, 8, 9).

## 3 Methylation biomarkers of cervical cancer-related genes

### 3.1 Host gene methylation biomarkers

Host gene methylation biomarkers have emerged as pivotal tools in the early detection and triage of cervical intraepithelial neoplasia (CIN) and cervical cancer, reflecting epigenetic alterations that accompany HPV-driven carcinogenesis. Among the most frequently studied methylation markers are PAX1, SOX1, ZNF582, CADM1, MAL, and miR-124-2, all of which exhibit hypermethylation in cervical cancer tissues and precancerous lesions, indicating their potential as diagnostic and prognostic biomarkers. For instance, PAX1 methylation has been extensively validated across diverse populations, demonstrating a sensitivity ranging from 70% to 85% for detecting CIN2+ lesions and a specificity exceeding 90%. This high specificity is crucial for minimizing false positives in screening programs. Moreover, PAX1 methylation status correlates synergistically with HPV16/18 infection, the most oncogenic HPV types, enhancing its predictive value for high-grade lesions and invasive cancer (10, 11).

The utility of combining multiple methylation markers has been shown to further improve diagnostic accuracy. Panels incorporating PAX1, SOX1, and ZNF582, for example, achieve area under the curve (AUC) values exceeding 0.90, reflecting excellent discrimination between high-grade lesions and benign or low-grade abnormalities. Such multigene methylation panels outperform single markers by capturing a broader spectrum of epigenetic changes associated with cervical carcinogenesis. This approach has been validated in various cohorts, including Chinese and Dutch populations, underscoring its robustness and cross-ethnic applicability (12, 11).

Beyond PAX1 and SOX1, other host genes such as CADM1 and MAL have also been implicated in cervical neoplasia. Meta-analyses reveal significantly higher promoter methylation frequencies of CADM1 and MAL in LSIL and HSIL than in normal controls, supporting their role as early indicators of neoplastic transformation (13). Similarly, miR-124-2 methylation has been associated with advanced transforming lesions and correlates with loss of HPV E4 expression, a marker of productive infection, suggesting that methylation changes reflect progression from productive to transforming HPV infections (14).

The clinical implementation of methylation testing benefits from its applicability to various sample types, including cervical scrapes, formalin-fixed paraffin-embedded (FFPE) biopsies, and even urine samples. Studies demonstrate consistent methylation detection in FFPE materials with adjusted cutoffs, facilitating retrospective analyses and integration into routine pathology workflows (15). Urine-based methylation assays targeting markers such as GHSR, SST, and ZIC1 show promise for non-invasive screening, particularly in populations with limited access to cervical sampling, with methylation levels correlating with disease severity (16, 17).

Mechanistically, methylation of these host genes often leads to transcriptional silencing of tumor suppressor functions, contributing to cervical carcinogenesis. For example, hypermethylation of PAX1 and SOX1 promoters results in decreased gene expression, which is associated with tumor progression and poorer prognosis (18, 15). Additionally, the methylation status of these genes may reflect underlying biological processes such as epithelial-mesenchymal transition (EMT), immune evasion, and viral oncogene activity, providing insights into tumor behavior and potential therapeutic targets (19, 20).

In summary, host gene methylation biomarkers, particularly PAX1, SOX1, and ZNF582, represent sensitive and specific molecular tools for cervical cancer screening and triage. Their integration into clinical practice, either as standalone tests or in combination with HPV genotyping and cytology, holds promise for improving early detection, risk stratification, and patient management, ultimately reducing cervical cancer incidence and mortality. Ongoing research aims to refine marker panels, validate their performance in diverse populations, and explore their utility in self-collected samples and post-treatment surveillance (4, 21, 22).

### 3.2 HPV viral gene methylation biomarkers

#### 3.2.1 HPV L1 and L2 gene methylation in viral integration and carcinogenic transformation

Methylation of HPV viral genes, particularly the late genes L1 and L2, has been increasingly recognized as a critical epigenetic event associated with viral integration into the host genome and the progression of cervical intraepithelial neoplasia (CIN) to cervical cancer. The L1 and L2 genes encode capsid proteins essential for viral assembly, and their methylation status reflects the viral life cycle and oncogenic transformation. Studies have demonstrated that HPV16 L1 methylation levels are significantly elevated in high-grade lesions, especially CIN3 and above, indicating a strong correlation with disease severity and malignant progression (23). Genome-wide methylation analyses revealed that specific CpG sites within the L1 and L2 regions exhibit progressive hypermethylation from normal tissue through high-grade squamous intraepithelial lesions (HSIL) to squamous cell carcinoma (SCC), supporting their role as biomarkers for lesion progression (23). Moreover, methylation of these late genes is thought to be linked to viral integration events, which disrupt the viral E2 gene and lead to deregulated expression of oncogenes E6 and E7, driving carcinogenesis. The hypermethylation of L1 and L2 may thus serve as a surrogate marker for viral integration and oncogenic transformation. In head and neck squamous cell carcinomas (HNSCC), similar patterns of HPV DNA methylation in L1 and L2 genes have been observed, with higher methylation levels correlating with tumor presence and grade, suggesting a conserved mechanism across HPV-associated cancers (24). These findings underscore the diagnostic and prognostic potential of HPV L1 and L2 methylation as biomarkers in cervical cancer screening and risk stratification.

#### 3.2.2 HPV E2/E4 gene methylation reflecting viral replication status and predicting lesion progression risk

The methylation status of HPV early genes E2 and E4 provides valuable insights into the viral replication state and the risk of lesion progression. The E2 gene plays a pivotal role in regulating viral transcription and replication, and its disruption through methylation or integration is a hallmark of oncogenic progression. Methylation of the E2 gene region can lead to loss of its regulatory function, resulting in unchecked expression of E6 and E7 oncogenes. Quantitative analyses of E2/E4 methylation have been utilized to assess viral activity; low methylation levels correspond to productive infections with active viral replication, whereas high methylation levels indicate transforming infections with increased oncogenic potential (25). Immunohistochemical studies combining E4 protein expression, a marker of productive infection, with methylation analyses have revealed that lesions exhibiting both E4 positivity and high methylation levels may represent a transitional state with a higher risk of progression, especially in immunocompromised populations such as women living with HIV. Furthermore, methylation of E2/E4 regions has been proposed as a predictive biomarker for lesion progression, aiding in the identification of patients who may benefit from closer surveillance or early intervention. The dynamic methylation changes in these early genes reflect the complex interplay between viral replication and host epigenetic regulation, highlighting their utility in refining cervical cancer screening algorithms.

#### 3.2.3 Combined viral and host methylation biomarker detection for dual validation and enhanced screening accuracy

Integrating viral gene methylation markers with host-cell methylation biomarkers offers a dual-validation approach that significantly enhances the accuracy and specificity of cervical cancer screening. Host gene methylation markers, such as FAM19A4, miR124-2, ZNF582, and ASCL1/LHX8, have demonstrated robust associations with high-grade cervical lesions and cancer, providing complementary information to viral methylation status. Combining these host markers with HPV viral gene methylation, particularly in the L1, L2, and E2/E4 regions, allows for a comprehensive assessment of both viral oncogenic activity and host epigenetic alterations. This combined strategy improves triage performance in HPV-positive women by reducing false positives and unnecessary colposcopy referrals while maintaining high sensitivity for detecting CIN3+ lesions. For example, the FAM19A4/miR124-2 methylation assay, when used alongside HPV16/18 genotyping and viral methylation markers, achieves superior discrimination of high-grade lesions compared to cytology alone. Additionally, studies have shown that methylation-negative results in both viral and host markers confer a low long-term risk of progression, providing reassurance and supporting extended screening intervals (26). This dual methylation biomarker approach is particularly valuable in populations with high HPV prevalence or immunosuppression, where conventional screening methods may be less effective (27). Overall, the integration of viral and host methylation markers represents a promising advancement in cervical cancer screening, enabling more precise risk stratification and personalized management of HPV-positive women.

## 4 Gene methylation detection technology

### 4.1 PCR-based detection methods

Polymerase chain reaction (PCR)-based methods have become pivotal in the detection of DNA methylation alterations associated with cervical cancer, offering a combination of sensitivity, specificity, and practicality that is suitable for clinical application. Among these, methylation-specific PCR (MSP) and its quantitative variant, real-time quantitative MSP (qMSP), are the most widely employed techniques due to their operational simplicity and cost-effectiveness. MSP involves bisulfite treatment of DNA, which converts unmethylated cytosines to uracils while leaving methylated cytosines unchanged, enabling the design of primers specific for methylated or unmethylated sequences. This specificity allows MSP to detect methylation status at particular gene loci with high accuracy. Real-time qMSP enhances this approach by quantifying methylation levels, providing a more nuanced assessment of methylation burden that correlates with disease severity. These methods have been successfully applied to cervical tissue and cervical smear samples, demonstrating robust sensitivity and specificity for detecting high-grade squamous intraepithelial lesions (HSIL) and squamous cell carcinoma (SCC). For instance, a five-gene methylation panel including FMN2, EDNRB, ZNF671, TBXT, and MOS, validated across diverse populations, achieved sensitivities up to 100% for SCC detection and specificities exceeding 90% in cervical tissue samples, with similarly high performance in cervical smears (28). This underscores the clinical utility of MSP/qMSP as a triage tool following high-risk HPV screening, enabling more precise identification of women requiring further diagnostic intervention or treatment.

The advancement of multiplex qMSP assays has further improved throughput and diagnostic efficiency by enabling simultaneous detection of multiple methylation markers. Multiplexing not only conserves sample material but also enhances diagnostic accuracy by combining markers with complementary methylation profiles. A notable example is the multiplex qMSP assay targeting PAX1, SOX1, and ZNF582, which has progressed into clinical trials. These genes exhibit promoter hypermethylation in cervical neoplasia, and their combined analysis improves discrimination between benign, precancerous, and malignant lesions. Clinical studies have demonstrated that such multiplex panels yield high sensitivity and specificity for CIN3+ lesions, with area under the curve (AUC) values of approximately 0.8–0.9, indicating strong diagnostic performance (29). The integration of multiplex qMSP assays into screening algorithms holds promise for reducing unnecessary colposcopies and overtreatment, particularly in resource-limited settings where cytology-based triage may be less feasible.

Restriction enzyme-PCR (RE-PCR) represents another PCR-based approach that enhances methylation detection specificity by exploiting methylation-sensitive restriction endonucleases. These enzymes selectively digest unmethylated DNA at specific recognition sites, leaving methylated DNA intact for subsequent PCR amplification. This enzymatic digestion step reduces background from unmethylated sequences, thereby increasing assay specificity. However, RE-PCR requires careful optimization of enzyme digestion conditions to ensure complete cleavage of unmethylated DNA without compromising methylated DNA integrity. The method’s reliance on enzyme efficiency and DNA quality can pose challenges in clinical samples, particularly those with limited or degraded DNA. Despite these limitations, RE-PCR has been applied successfully to cervical cancer methylation studies, providing an alternative or complementary approach to bisulfite-based MSP methods (30). Its utility may be enhanced when combined with quantitative PCR techniques or multiplexing to improve sensitivity and throughput.

Overall, PCR-based methylation detection methods offer a balance of sensitivity, specificity, and practicality that supports their clinical translation in cervical cancer screening and triage. MSP and qMSP are particularly well-suited for routine use due to their simplicity and cost-effectiveness, while multiplex qMSP assays improve diagnostic accuracy and efficiency by targeting multiple methylation markers simultaneously. RE-PCR adds specificity through enzymatic digestion but requires meticulous optimization. The ongoing validation of methylation marker panels across diverse populations and sample types, including cervical smears, supports the potential of these PCR-based methods to complement or even replace cytology in HPV-positive women, facilitating earlier and more accurate identification of high-risk lesions and reducing unnecessary interventions. Future developments may focus on integrating these assays into automated platforms and combining methylation analysis with other molecular markers to further enhance cervical cancer screening programs worldwide (28, 29, 30).

### 4.2 Sequencing-based detection methods

Sequencing-based methods have become pivotal in the detection and quantification of DNA methylation alterations associated with cervical cancer, offering high-resolution, comprehensive profiling capabilities. Among these, bisulfite sequencing PCR (BSP) remains the gold standard for methylation analysis at single-CpG-site resolution. BSP involves the treatment of DNA with sodium bisulfite, which converts unmethylated cytosines to uracils while leaving methylated cytosines unchanged, enabling subsequent sequencing to distinguish methylated from unmethylated cytosines. This method allows for quantitative assessment of methylation levels at individual CpG sites, providing precise epigenetic information critical for understanding gene regulation in cervical carcinogenesis. However, BSP is labor-intensive, technically demanding, and costly, limiting its scalability for large numbers of samples or genome-wide analyses (30). The bisulfite treatment itself can cause DNA degradation, further complicating sample preparation and reducing DNA yield, which is particularly challenging when working with limited clinical specimens (31).

To overcome these limitations, next-generation sequencing (NGS) technologies have been increasingly applied to methylation analysis in cervical cancer. Targeted methylation sequencing enables simultaneous interrogation of hundreds of genes or specific genomic regions of interest, facilitating the discovery and validation of methylation biomarkers for early detection and prognosis. Whole-genome bisulfite sequencing (WGBS), another NGS-based approach, provides an unbiased, base-resolution map of methylation across the entire genome, capturing both known and novel epigenetic alterations. These high-throughput methods have been instrumental in identifying differential methylation patterns associated with cervical intraepithelial neoplasia progression and invasive carcinoma, revealing epigenetic drivers and potential therapeutic targets. For example, genome-wide methylation profiling has uncovered hypermethylation of tumor suppressor genes such as NRG1, whose promoter methylation correlates with decreased expression and cervical cancer progression (32). Moreover, integrated analyses combining methylation data with transcriptomic and immune infiltration profiles have elucidated the complex interplay between epigenetic modifications and the tumor microenvironment, informing immunotherapy strategies (33). Despite their advantages, NGS-based methylation assays require sophisticated bioinformatics pipelines and remain relatively costly, which may limit their routine clinical application in resource-constrained settings.

Emerging third-generation sequencing technologies, including single-molecule real-time (SMRT) sequencing and nanopore sequencing, offer novel opportunities for direct detection of DNA methylation without the need for bisulfite conversion. SMRT sequencing, developed by Pacific Biosciences, detects methylation by measuring the kinetics of DNA polymerase during sequencing, allowing for the identification of modified bases in native DNA molecules. Similarly, nanopore sequencing, exemplified by Oxford Nanopore Technologies, reads DNA sequences by monitoring changes in electrical current as single DNA strands pass through a nanopore, enabling simultaneous detection of sequence and epigenetic modifications. These approaches reduce DNA damage and loss associated with bisulfite treatment, preserve DNA integrity, and allow for longer read lengths, which facilitate the analysis of methylation patterns in repetitive or complex genomic regions. In cervical cancer research, these technologies hold promise for comprehensive methylation profiling and biomarker discovery with improved accuracy and efficiency. However, challenges remain, including higher error rates compared to short-read sequencing and the need for optimized algorithms to accurately call methylation states. As these technologies mature, they are expected to complement existing methods and enhance the epigenetic characterization of cervical cancer (33).

In summary, sequencing-based methylation detection methods have evolved from laborious, locus-specific BSP to high-throughput NGS and direct methylation detection via third-generation sequencing. BSP provides precise quantification at single-CpG resolution but is limited by complexity and cost. NGS approaches enable broad, multiplexed methylation analysis suitable for biomarker discovery and validation, while SMRT and nanopore sequencing offer direct, bisulfite-free methylation detection with potential advantages in DNA preservation and long-read capabilities. Together, these methods contribute to advancing cervical cancer screening by enabling detailed epigenetic profiling, improving early diagnosis, risk stratification, and informing personalized therapeutic interventions. However, third-generation sequencing technologies also face significant challenges in clinical applications. Firstly, their single-base sequencing error rate remains significantly higher than that of short-read sequencing (NGS), which can affect the accuracy of CpG site methylation determination. Secondly, the high sequencing costs and complex bioinformatics analysis algorithms make these technologies difficult to use as a routine options for large-scale primary screening of cervical cancer at this stage. Several studies have systematically compared these sequencing-based methods. For example, Kurdyukov and Bullock (2016) reviewed the strengths and limitations of BSP, NGS, and third-generation sequencing, highlighting trade-offs in resolution, throughput, cost, and DNA integrity (30). More recent comparisons have demonstrated that while NGS offers high multiplexing capacity, third-generation platforms reduce bisulfite-induced bias and enable long-read methylation profiling (31). These comparative analyses inform the choice of method for specific screening or biomarker discovery purposes.

### 4.3 Other emerging detection technologies

#### 4.3.1 Mass spectrometry (e.g., MALDI-TOF MS) for high-throughput quantitative methylation analysis with high precision but expensive equipment

Mass spectrometry-based methods, particularly matrix-assisted laser desorption/ionization time-of-flight mass spectrometry (MALDI-TOF MS), have emerged as powerful tools for high-throughput and quantitative analysis of DNA methylation. These technologies enable precise measurement of methylation levels at multiple CpG sites simultaneously, offering a comprehensive methylation profile that is crucial for understanding epigenetic alterations in cervical cancer (CC). MALDI-TOF MS operates by ionizing DNA fragments and measuring their mass-to-charge ratios, allowing differentiation between methylated and unmethylated cytosines after bisulfite conversion and PCR amplification. The high sensitivity and specificity of MALDI-TOF MS facilitate the detection of subtle methylation changes that may serve as early biomarkers for CC screening and prognosis. For instance, genome-wide methylation analyses using high-throughput platforms have identified numerous differentially methylated genes and long non-coding RNAs (lncRNAs) implicated in CC pathogenesis, such as ZNF471 and SOX21-AS1, whose methylation status correlates with tumor progression and patient survival (34, 35). Despite its advantages, the widespread clinical application of MALDI-TOF MS is limited by the high cost of instrumentation and the need for specialized technical expertise. The initial investment and maintenance expenses restrict its availability primarily to well-equipped research centers and tertiary hospitals, posing challenges for implementation in low-resource settings where CC burden is often highest (36). Nevertheless, MALDI-TOF MS remains a gold standard for validating methylation biomarkers discovered through array-based or sequencing approaches, providing robust quantitative data that underpin the development of diagnostic assays.

#### 4.3.2 Droplet digital PCR for absolute quantification of methylated DNA suitable for low-abundance samples such as self-collected specimens

Droplet digital PCR (ddPCR) has revolutionized the detection and quantification of DNA methylation by enabling absolute quantification of methylated DNA molecules without the need for standard curves. This technology partitions a DNA sample into thousands of nanoliter-sized droplets, each acting as an individual PCR reaction, allowing precise quantification of target molecules. ddPCR is particularly advantageous for analyzing low-abundance methylated DNA in clinical specimens, including self-collected samples such as vaginal swabs or urine, which are increasingly recognized for their potential in CC screening programs aimed at improving accessibility and compliance (37). The high sensitivity of ddPCR facilitates detection of rare methylation events associated with early neoplastic changes, such as methylation of tumor suppressor genes (e.g., PAX1, ZSCAN18) or oncogenes, which have been shown to correlate with cervical intraepithelial neoplasia and invasive cancer (38, 39). Moreover, ddPCR’s quantitative output allows for monitoring of methylation dynamics over time, offering utility in treatment response assessment and disease surveillance. The method’s compatibility with minimal DNA input and its tolerance to sample impurities make it well-suited for non-invasive or minimally invasive sample types. However, ddPCR requires specialized equipment and reagents, and assay design must be carefully optimized to distinguish methylated from unmethylated sequences, often involving methylation-specific primers or probes. Despite these challenges, ddPCR is gaining traction as a clinically applicable tool for methylation-based CC screening, particularly in decentralized or resource-limited settings where self-sampling could expand screening coverage.

#### 4.3.3 Microfluidic chip and electrochemical sensor technologies under development for point-of-care testing, suitable for primary healthcare settings

Innovative microfluidic chip platforms and electrochemical sensor technologies are being actively developed to enable rapid, sensitive, and cost-effective detection of DNA methylation at the point-of-care (POCT). These emerging technologies aim to overcome the limitations of conventional laboratory-based assays by integrating sample processing, target detection, and signal readout into compact, user-friendly devices suitable for use in primary healthcare or low-resource environments. Microfluidic chips leverage miniaturized fluidic channels and chambers to perform automated bisulfite conversion, PCR amplification, and methylation-specific detection with minimal reagent consumption and reduced assay time. For example, microfluidic methylation assays have demonstrated the capacity to detect methylation biomarkers relevant to CC with high sensitivity and specificity, facilitating early diagnosis and triage (40). Electrochemical sensors utilize methylation-sensitive probes immobilized on electrode surfaces to transduce methylation events into measurable electrical signals. These sensors offer advantages such as portability, low cost, and rapid response, making them attractive for screening programs in community clinics or remote areas. Recent advances have improved their analytical performance, enabling detection of methylated DNA in complex biological matrices, including cervical swabs and plasma (41). The development of integrated POCT devices combining microfluidics and electrochemical sensing holds promise for real-time methylation analysis, potentially allowing immediate clinical decision-making. However, challenges remain in standardizing assay protocols, ensuring reproducibility, and validating clinical utility across diverse populations. Continued research and technological refinement are essential to translate these platforms into routine CC screening tools, particularly to address disparities in healthcare access and improve early detection rates globally. Although ddPCR excels in absolute quantification and detection of low-abundance samples, its limited throughput makes it difficult to achieve high-throughput multiplex detection of multiple targets in a single reaction. Constrained by the physical limits of droplet generation and reading, its sample processing efficiency for large-scale population screening is far lower than that of real-time fluorescent quantitative PCR platforms.

## 5 Clinical application of gene methylation detection in cervical cancer screening

### 5.1 As a triage tool for HPV-positive patients

The application of DNA methylation detection as a triage tool for HPV-positive women represents a significant advancement in cervical cancer screening, addressing the limitations of HPV testing alone. While high-risk HPV (hrHPV) testing is highly sensitive for identifying women at risk of cervical intraepithelial neoplasia (CIN) and cervical cancer, it lacks specificity, leading to unnecessary colposcopy referrals and overtreatment. Methylation assays targeting host-cell genes have emerged as effective molecular triage methods to stratify risk among HPV-positive women, thereby optimizing clinical management by identifying those who require immediate colposcopic evaluation and reducing unnecessary procedures in low-risk individuals.

Several studies have demonstrated the utility of methylation markers in this context. For instance, a five-gene methylation panel including FMN2, EDNRB, ZNF671, TBXT, and MOS showed high sensitivity and specificity in detecting squamous cell carcinoma (SCC) and high-grade squamous intraepithelial lesions (HSIL) in cervical smears from diverse populations, including the USA, South Africa, and Vietnam. This panel achieved a sensitivity of 87% and a specificity of 95% for SCC detection, with an area under the curve (AUC) of 0.925, and 70% sensitivity and 94% specificity for HSIL detection (AUC = 0.884), outperforming HPV testing alone (28). These findings underscore the potential of methylation markers to accurately identify high-risk lesions among HPV-positive women, facilitating timely treatment and reducing unnecessary colposcopy referrals.

Among individual methylation markers, PAX1 methylation has been extensively studied and validated as a triage tool. Quantitative methylation-specific PCR assays have shown that PAX1 methylation levels increase with the severity of cervical lesions and that their diagnostic performance is superior to the of HPV testing and cytology. In a study comparing PAX1 methylation with folic acid receptor-mediated diagnosis, ThinPrep cytology, and HPV testing, PAX1 methylation exhibited the highest area under the curve (AUC = 0.902), with sensitivity and specificity of 92.3% and 78.6%, respectively, for invasive cervical cancer detection (10). Furthermore, in HPV-positive women with non-HPV16/18 high-risk HPV infections, PAX1 methylation demonstrated higher specificity and positive predictive value (PPV) for CIN2+/CIN3+ lesions compared with cytology, with AUCs of 0.87 for both CIN2+ and CIN3+ detection (42). These data highlight PAX1 methylation as a robust biomarker for triaging HPV-positive women, especially in cases where cytology may be less informative.

Methylation testing also offers dynamic monitoring capabilities. DNA methylation changes can reflect the natural history of HPV infection and cervical lesion progression or regression. For example, methylation markers such as ASCL1/LHX8 and FAM19A4/miR124-2 have been evaluated for post-treatment surveillance, showing high sensitivity for detecting recurrent CIN2/3 lesions. Methylation levels were significantly higher in women with persistent HPV infections compared to those with incident or no HPV infections, indicating that methylation assays can guide follow-up intervals and identify women at risk of recurrence who require re-treatment (22). This dynamic aspect of methylation testing enhances its clinical utility beyond initial triage, supporting personalized management strategies.

Large-scale population-based studies have further validated methylation-based triage approaches. The WID-qCIN test, which assesses methylation of genes DPP6, RALYL, and GSX1, combined with HPV16/18 genotyping, detected 93.4% of CIN3 and 100% of invasive cervical cancers in a cohort of over 28,000 women. This combination predicted 69.4% of incident CIN2+ cases compared with 18.2% predicted by cytology, with fewer colposcopy referrals required per detected case, demonstrating improved efficiency and accuracy over traditional triage methods (37). Similarly, the S5 DNA methylation classifier, targeting EPB41L3 and HPV gene regions, showed high sensitivity (93%) for prevalent CIN3 and invasive cervical cancer, outperforming HPV16/18 genotyping alone, and predicted future CIN3+ development up to 5 years before diagnosis (43). These findings support the integration of methylation markers into screening algorithms to enhance risk stratification and early detection.

The prognostic value of methylation markers has also been explored. For example, ZNF671 methylation measured in formalin-fixed paraffin-embedded cervical tissue correlated with lesion progression and was more frequently detected in progressive and recurrent CIN than in regressive or persistent lesions. Patients with ZNF671 methylation positivity, especially those over 30 years or with HPV16 infection, were more likely to experience disease progression, suggesting that methylation status can inform surveillance intensity and therapeutic decisions (44). This prognostic capability complements the triage function, enabling more nuanced patient management.

Mechanistically, DNA methylation reflects epigenetic alterations induced by persistent hrHPV infection and host cellular responses. Methylation levels of specific tumor suppressor genes and imprinted regions increase with lesion severity, correlating with histopathological grades from CIN1 to invasive cancer. For instance, methylation of PAX1 and ZNF582 correlates with p16/Ki67 immunoscores, which are established markers of lesion severity, indicating that methylation assays capture underlying biological changes associated with neoplastic progression (45). Moreover, methylation changes can be detected in various sample types, including cervical smears, urine, and tissue biopsies, facilitating flexible and minimally invasive screening approaches (46).

In summary, DNA methylation detection serves as an effective triage tool for HPV-positive women by accurately identifying those at high risk for CIN2+ and cervical cancer who require colposcopy referral, while reducing unnecessary procedures in low-risk individuals. Markers such as PAX1, ZNF671, and multigene panels have demonstrated superior diagnostic performance compared to cytology and HPV genotyping alone. Additionally, methylation assays enable dynamic monitoring of lesion progression or regression, supporting personalized follow-up strategies. The integration of methylation testing into cervical cancer screening programs promises to enhance early detection, optimize resource utilization, and improve patient outcomes globally, particularly in low-resource settings where cytology infrastructure is limited. Ongoing validation and standardization efforts will further solidify the role of methylation markers as indispensable tools in the management of HPV-positive women.

### 5.2 Application in self-sampling screening

Self-sampling methods, including vaginal swabs and urine samples, have emerged as promising approaches to increase cervical cancer screening coverage, particularly in remote areas and among women reluctant to undergo traditional gynecological examinations. These methods address significant barriers to screening participation by offering a more accessible, less invasive, and privacy-preserving alternative, which is crucial for improving early detection rates in underserved populations. It must also be clearly recognized that urine samples contain extremely low levels of DNA and are subject to significant dilution effects and DNA degradation issues. These biochemical characteristics often result in lower clinical sensitivity for urine methylation testing compared with that of tissue or cervical scrapings, requiring careful evaluation of its applicability during clinical translation. The integration of DNA methylation biomarker detection into self-sampling protocols has demonstrated considerable potential, as methylation markers exhibit robust stability in self-collected specimens and maintain diagnostic performance comparable to that observed in clinician-collected samples.

Multiple studies have underscored the efficacy of methylation markers such as SOX1 and PAX1 in self-sampled materials. For instance, SOX1 promoter hypermethylation has been shown to have a sensitivity of approximately 75% for detecting high-grade cervical intraepithelial neoplasia (CIN3+) in self-collected samples, closely paralleling the performance observed in physician-collected specimens (47). This high sensitivity, coupled with acceptable specificity, supports the utility of SOX1 methylation as a reliable biomarker in non-invasive screening contexts. Moreover, meta-analyses have reinforced the diagnostic value of methylation markers in urine samples, highlighting their potential as effective non-invasive tools for cervical cancer screening (48).

The stability of methylation markers in self-collected samples is a critical advantage, as DNA methylation patterns are less susceptible to degradation and sample variability compared to other molecular markers. This robustness facilitates accurate detection even when sample collection and handling conditions are suboptimal, which is often the case in community-based or remote screening programs. Additionally, the use of quantitative methylation-specific PCR (qMSP) and methylation-specific PCR (MSP) techniques enables sensitive and specific detection of methylated DNA in these samples, further enhancing diagnostic accuracy (48).

Implementing self-sampling combined with methylation testing can substantially reduce logistical and human resource burdens associated with traditional screening methods. By enabling women to collect samples at home or in community settings, the need for clinical infrastructure and trained personnel is diminished, which is particularly beneficial in low-resource environments. This decentralization of screening promotes community-level engagement and can lead to higher screening uptake and earlier detection of precancerous lesions and cervical cancer (49).

Furthermore, cohort studies have demonstrated that methylation-based triage strategies applied to self-sampled specimens can improve risk stratification among high-risk human papillomavirus (hrHPV)-positive women. For example, SOX1/PAX1 methylation testing has shown sensitivity and specificity superior to those of cytology for triaging hrHPV-positive individuals, enabling more efficient identification of women requiring colposcopy and reducing unnecessary referrals (50). This enhanced triage capability is particularly valuable in self-sampling programs, where cytological evaluation is not feasible.

The combination of self-sampling and methylation marker detection also aligns with the global movement toward community-based cervical cancer screening models. By lowering barriers to access and streamlining diagnostic workflows, this approach supports the World Health Organization’s goals for cervical cancer elimination, especially in regions with limited healthcare infrastructure. Moreover, the cost-effectiveness of methylation assays, when integrated with self-sampling, may facilitate sustainable screening programs by reducing expenses related to sample collection, transportation, and clinical visits (49).

In summary, the application of DNA methylation detection in self-sampling screening represents a significant advancement in cervical cancer prevention. The high stability and diagnostic performance of methylation markers such as SOX1 and PAX1 in self-collected vaginal and urine samples provide a reliable alternative to clinician-collected specimens. This strategy not only enhances screening coverage and accessibility but also optimizes resource utilization by enabling community-level screening and efficient triage of hrHPV-positive women. Continued research and validation in diverse populations will further consolidate the role of methylation-based self-sampling screening in global cervical cancer control efforts (47–50).

### 5.3 Value in post-treatment follow-up

Cervical conization, including procedures such as loop electrosurgical excision procedure (LEEP), is a common therapeutic intervention for cervical intraepithelial neoplasia (CIN) and early-stage cervical cancer (CC). However, patients undergoing conization remain at a significant risk of residual disease and recurrence, necessitating vigilant post-treatment surveillance. DNA methylation detection has emerged as a promising molecular tool to enhance the sensitivity and specificity of post-treatment monitoring, offering advantages over traditional cytology-based follow-up.

Firstly, the risk of recurrence after cervical conization is notably high, especially in cases with positive resection margins or high-grade lesions. Studies have demonstrated that methylation markers, such as those targeting tumor suppressor genes FAM19A4 and miR124-2, can detect residual or recurrent lesions earlier than cytology or HPV testing alone. For instance, clearance of high-risk HPV (hrHPV) and methylation negativity shortly after conization correlate strongly with the absence of residual disease, with a positive predictive value of up to 95% for no residual cancer, indicating that methylation testing can serve as a reliable biomarker for treatment success and risk stratification (51). This early detection capability is critical because residual or recurrent disease, if identified promptly, can be managed effectively to prevent progression.

Secondly, the dynamics of methylation levels post-treatment provide valuable prognostic information. A significant decline in methylation levels of specific genes post-conization suggests effective eradication of neoplastic cells, whereas persistently high methylation levels are indicative of residual disease or an increased risk of recurrence. The sensitivity of methylation assays in detecting residual or recurrent lesions has been reported to exceed 80%, outperforming cytology in many settings (51). For example, methylation of the ZNF671 promoter has been shown to be a sensitive marker for residual disease, with higher methylation correlating with poor prognosis and recurrence risk. Similarly, methylation signatures involving genes such as PAX1 and SOX1 have been associated with treatment outcomes and survival, underscoring their utility in post-therapeutic surveillance.

Thirdly, methylation-based testing can reduce the need for repeated cytological examinations, which are often associated with patient anxiety, discomfort, and increased healthcare resource utilization. Unlike cytology, which requires skilled personnel and may suffer from interobserver variability, methylation assays provide objective, reproducible molecular readouts that can be obtained from minimally invasive samples such as cervical cytology or even urine (48). This molecular approach streamlines follow-up protocols, potentially decreasing the frequency of visits and invasive procedures without compromising diagnostic accuracy. Moreover, methylation testing can be integrated with HPV genotyping to further refine risk assessment, as demonstrated by combined assays detecting methylation markers alongside HPV16/18 genotypes, which have shown superior predictive performance for high-grade lesions and invasive cancer (37).

Furthermore, the application of methylation detection in post-treatment follow-up aligns with the evolving paradigm of personalized medicine. By stratifying patients based on methylation profiles, clinicians can tailor surveillance intensity and therapeutic interventions, focusing resources on high-risk individuals while sparing low-risk patients from unnecessary procedures. This stratification is particularly relevant in low- and middle-income countries where healthcare resources are limited, and efficient allocation is paramount (36).

In addition to clinical utility, methylation markers have demonstrated biological relevance in cervical carcinogenesis and treatment response. Aberrant methylation of tumor suppressor genes contributes to oncogenic transformation and progression, and its reversal post-treatment reflects tumor clearance. For example, the methylation status of genes such as ZNF471 and PAX1 not only serves as a biomarker but also correlates with tumor suppressor functions and therapeutic sensitivity. This dual role enhances the credibility of methylation assays as surrogate endpoints in clinical follow-up.

In summary, DNA methylation detection offers significant value in the post-treatment follow-up of patients who have undergone cervical conization by enabling early identification of residual or recurrent disease, providing prognostic insights through methylation dynamics, and reducing reliance on repeated cytological examinations. Its high sensitivity (>80%) and specificity, combined with the potential to integrate with HPV genotyping, position methylation testing as a superior alternative or adjunct to conventional surveillance methods. Adoption of methylation-based follow-up protocols can improve patient outcomes, optimize healthcare resource utilization, and facilitate personalized management strategies in cervical cancer care.

## 6 Clinical performance evaluation of gene methylation detection

### 6.1 Sensitivity and specificity analysis

Host gene methylation detection has emerged as a promising biomarker for cervical intraepithelial neoplasia grade 2 or worse (CIN2+), with reported sensitivities ranging from 70% to 90% and specificities between 85% and 95%, outperforming traditional cytological screening, whose sensitivity typically ranges from 50% to 70% (3). This superior diagnostic performance is attributed to the progressive increase in methylation levels of specific tumor suppressor genes as cervical lesions advance in severity, enabling more accurate discrimination between benign and precancerous or cancerous states. For instance, assays such as the GynTect^®^ panel targeting genes, including ASTN1, DLX1, ITGA4, RXFP3, SOX17, and ZNF671, have demonstrated high specificity and sensitivity for detecting CIN2+ and CIN3+ lesions, with odds ratios indicating strong associations between methylation positivity and lesion severity (52). Moreover, methylation markers such as ASCL1/LHX8 and FAM19A4/miR124-2 have shown excellent performance in post-treatment surveillance, detecting recurrent CIN2/3 with sensitivities exceeding 90% for CIN3 recurrence, highlighting their utility beyond initial screening (22).

The use of multigene methylation panels further enhances sensitivity, often achieving detection rates above 90% for CIN2+ lesions. For example, the S5 classifier, which combines methylation markers from both host and viral genes (including EPB41L3 and HPV L1/L2 regions), has demonstrated sensitivities up to 93% for CIN3+ detection, albeit with some reduction in specificity to approximately 50%–60% (43). This trade-off between sensitivity and specificity necessitates careful consideration of screening goals; prioritizing sensitivity may reduce missed high-grade lesions but increase false positives and subsequent colposcopy referrals, whereas emphasizing specificity may limit unnecessary procedures but risk under-detection. Studies have suggested that combining methylation testing with HPV genotyping or cytology can optimize this balance, improving positive predictive values and reducing overtreatment (53, 54).

Viral DNA methylation analysis, particularly targeting high-risk HPV types such as HPV16, HPV18, HPV31, and HPV33, offers complementary diagnostic value. Viral methylation markers typically exhibit sensitivities of 60% to 80% for detecting CIN3+ lesions, with specificities exceeding 90%, making them suitable as adjunctive biomarkers to host gene methylation assays (3, 55). For instance, methylation levels in HPV31 lineages correlate with oncogenic potential and lesion severity, supporting their role in risk stratification (55). The integration of viral methylation markers into multigene panels such as S5 enhances predictive accuracy for high-grade lesions and invasive cervical cancer, facilitating early intervention (43).

Emerging technologies also explore non-invasive sampling methods such as urine-based methylation testing, which has shown promising sensitivity (approximately 86%) and specificity (approximately 70%) for CIN3+ detection, potentially increasing screening coverage among women less likely to participate in traditional screening programs (46). Additionally, machine learning models incorporating methylation data from multiple genes have achieved area under the curve (AUC) values up to 0.90 for CIN2+ detection, outperforming HPV testing and cytology used alone, and offering personalized risk assessment (56).

### 6.2 Comparison with existing screening methods

The application of gene methylation detection in cervical cancer screening offers several advantages over traditional methods such as HPV testing and cytology, addressing some of their inherent limitations. Compared to HPV detection, methylation assays demonstrate significantly higher specificity, which is crucial for reducing unnecessary referrals for colposcopy. High-risk HPV (hrHPV) testing, while sensitive, often lacks specificity because it cannot distinguish between transient infections and clinically significant lesions requiring intervention. This limitation results in a substantial number of false-positive cases, leading to over-referral and overtreatment. Studies comparing commercially available methylation-specific PCR assays, such as GynTect^®^ and the QIAsure Methylation Test, have shown that methylation tests can detect all cases of cervical carcinoma and carcinoma *in situ* with high sensitivity while markedly reducing false-positive rates. For instance, the GynTect^®^ assay exhibited a false-positive rate as low as 5.7% in hrHPV-positive women with no cytological abnormalities, compared with 26.4% for the QIAsure test, indicating superior specificity (57). This enhanced specificity translates into a potential reduction of 50%–70% in colposcopy referrals, alleviating the burden on healthcare systems and minimizing patient anxiety and morbidity associated with unnecessary invasive procedures.

Compared with cytology, methylation detection offers greater objectivity and reproducibility. Cytological evaluation is inherently subjective, relying heavily on the expertise and experience of cytopathologists, which can lead to interobserver variability and inconsistent results. Moreover, cytology requires well-trained personnel and is labor-intensive, limiting its scalability and automation potential. Methylation assays, by contrast, are molecular tests that provide quantitative and objective results, minimizing subjective interpretation. The FAM19A4/miR124-2 methylation test, evaluated in a large European multicenter study involving 2,384 HPV-positive samples, demonstrated consistent sensitivity and specificity across different centers, sample collection media, DNA extraction methods, and HPV screening tests, underscoring its robustness and suitability for automated high-throughput screening (58). This objectivity and adaptability make methylation testing a promising alternative or adjunct to cytology, especially in settings where cytological expertise is limited.

Furthermore, combining HPV testing with methylation-based triage strategies significantly improves the positive predictive value (PPV) for detecting high-grade cervical lesions. While standalone hrHPV testing yields a PPV of approximately 10%–20%, the integration of methylation markers as a reflex test for HPV-positive women can elevate the PPV to 50%–70%. This improvement is clinically meaningful as it enhances the precision of identifying women who truly harbor precancerous or cancerous lesions, thereby optimizing patient management and resource allocation. For example, the S5 methylation classifier, which incorporates methylation markers from HPV and host genes, has demonstrated high sensitivity and specificity for CIN3 and cervical cancer detection across diverse geographic and economic settings. At an optimized cutoff, the S5 test achieved a sensitivity of 95.77% and improved specificity compared to HPV16/18 genotyping alone, highlighting its potential as both a screening and triage tool (59). Such combined strategies leverage the high sensitivity of HPV testing and the high specificity of methylation assays, creating a balanced approach that minimizes both missed diagnoses and unnecessary interventions.

In addition to diagnostic performance, methylation testing offers practical advantages, including the potential for automation and standardization, which are critical for large-scale screening programs. The molecular nature of methylation assays allows for integration into automated platforms, reducing human error and variability. This contrasts with cytology, where manual slide reading remains a bottleneck. Moreover, methylation markers are stable and can be detected in various sample types, including cervical scrapes and self-collected specimens, facilitating broader screening coverage and accessibility.

In summary, gene methylation detection in cervical cancer screening surpasses existing methods by providing higher specificity than HPV testing, reducing unnecessary colposcopy referrals by 50%–70%, and offering objective, reproducible results that are less dependent on subjective interpretation compared to cytology. The combined use of HPV testing followed by methylation-based triage significantly enhances the positive predictive value, reaching 50%–70%, which is substantially better than HPV testing alone. These advantages position methylation assays as a valuable tool in the evolving landscape of cervical cancer screening, with the potential to improve clinical outcomes, optimize resource utilization, and expand screening accessibility worldwide. Continued validation and implementation studies are warranted to fully integrate methylation testing into routine clinical practice.

## 7 Challenges and solutions in gene methylation detection

### 7.1 Standardization and quality control

The application of DNA methylation detection in cervical cancer screening has shown promising potential as a triage tool for high-risk human papillomavirus (hrHPV)-positive women, offering improved specificity over hrHPV testing alone. However, a significant challenge limiting the widespread clinical adoption and comparability of methylation assays is the lack of standardization across studies and platforms. Different research groups have employed diverse methylation markers, detection platforms, and threshold values, resulting in considerable variability in diagnostic performance and complicating cross-study comparisons. For instance, commercially available assays such as GynTect^®^ and QIAsure Methylation Test utilize distinct panels of methylation markers and exhibit differences in sensitivity and specificity for detecting cervical intraepithelial neoplasia (CIN) grades and cervical cancer, with GynTect^®^ generally demonstrating higher specificity in hrHPV-positive populations (57). Similarly, the S5 classifier, which combines methylation of host and HPV genes, has shown robust predictive power for CIN3+ lesions but requires consistent assay conditions and cutoff adjustments when applied to different sample types such as formalin-fixed paraffin-embedded (FFPE) biopsies versus exfoliated cervical cells (15). This heterogeneity in assay design and interpretation thresholds underscores the urgent need for internationally recognized reference standards and quality control (QC) systems.

Establishing such standards would involve the development and distribution of methylation reference materials, including well-characterized methylation-positive and -negative controls, to calibrate assay performance and ensure reproducibility across laboratories. For example, targeted bisulfite next-generation sequencing (NGS) platforms have been employed to validate novel methylation markers such as CA10, DPP10, FMN2, and HAS1, demonstrating consistent methylation patterns correlating with disease progression from normal epithelium through CIN grades to invasive cancer (60). Incorporating these validated markers into standardized panels and using uniform data analysis pipelines would enhance comparability. Moreover, harmonizing positivity threshold settings is critical; studies have shown that adjusting cutoffs can balance sensitivity and specificity to optimize clinical utility, as demonstrated by variations in the S5 classifier’s performance according to DNA conversion efficiency and sample type (15).

In addition to assay standardization, rigorous quality control measures must be implemented throughout the testing workflow. This includes standardized protocols for sample collection, storage, DNA extraction, bisulfite conversion, and methylation detection to minimize pre-analytical variability. For instance, the performance of methylation markers such as ZNF671 has been validated in FFPE samples, demonstrating prognostic value for progressive CIN lesions; however, differences between FFPE and cervical scrape samples necessitate careful methodological consistency (44). Furthermore, inter-laboratory proficiency testing and external quality assessment schemes should be established to monitor assay accuracy and reproducibility over time.

Clinical translation of methylation-based assays requires validation in large-scale, prospective cohort studies to confirm their stability, reproducibility, and predictive value in diverse populations. Several studies have highlighted the need for such validation; for example, the WID-CIN test, a DNA methylation signature comprising 5,000 CpG sites, demonstrated high sensitivity and specificity for CIN3+ detection in independent diagnostic and predictive validation cohorts, underscoring the importance of large, well-characterized sample sets for robust assay evaluation (61). Similarly, methylation markers have shown promise in triaging hrHPV-positive women in various settings, including low- and middle-income countries (LMICs) and HIV-positive populations, but require further prospective validation to ensure consistent performance.

Moreover, the integration of methylation testing into clinical workflows must consider the balance between sensitivity and specificity to minimize unnecessary colposcopy referrals and overtreatment. Meta-analyses have confirmed the potential of methylation markers as triage tools with pooled sensitivities of approximately 68%–78% and specificities of approximately 74%–75% for detecting CIN2+ and CIN3+ lesions, but also emphasize the need for standardized protocols to achieve these performance metrics reliably (62). The development of consensus guidelines on assay selection, interpretation criteria, and clinical decision thresholds will be essential to facilitate the adoption of methylation testing in cervical cancer screening programs.

In summary, the advancement of DNA methylation detection in cervical cancer screening hinges on the establishment of international standards and quality control frameworks that address the current heterogeneity in methylation markers, detection platforms, and threshold definitions. Standardized reference materials, harmonized protocols, and large-scale prospective validations are critical to ensuring assay stability, reproducibility, and clinical utility. These efforts will enable methylation assays to fulfill their promise as effective, reliable triage tools that complement hrHPV testing, ultimately improving early detection and management of cervical precancerous lesions and cancer.

### 7.2 Sample processing and DNA quality

The process of sample handling and DNA quality preservation is critical in the application of DNA methylation assays for cervical cancer screening, as these factors directly influence the sensitivity and reliability of detecting methylation markers, especially in low-abundance samples. One of the major challenges in methylation analysis is the bisulfite treatment of DNA, which converts unmethylated cytosines to uracils, leaving methylated cytosines unchanged. This chemical conversion, while essential for distinguishing methylated from unmethylated DNA, inherently causes DNA degradation and loss, which can significantly impair the detection sensitivity in samples with limited DNA quantities, such as self-collected or minimally invasive specimens. Studies have demonstrated that bisulfite treatment can lead to fragmentation and reduced DNA yield, thereby affecting downstream methylation analyses and potentially leading to false-negative results in low-input samples (63).

To mitigate these issues, optimization of DNA extraction and bisulfite conversion protocols has been a focus of recent research. The use of protective agents during bisulfite treatment, such as DNA stabilizers or antioxidants, can reduce DNA degradation by minimizing oxidative damage and depurination events. Additionally, reducing the duration and harshness of bisulfite exposure without compromising conversion efficiency has been shown to improve DNA integrity and yield. For instance, direct bisulfite conversion protocols applied to cervical samples have been developed to streamline workflows and enhance sample utilization by eliminating separate DNA isolation steps. Such protocols have demonstrated high technical and analytical performance, with methylation results strongly correlated with those obtained using conventional methods, indicating their potential to improve sample throughput and reduce DNA loss (63).

Moreover, the preservation and transport conditions of self-sampling specimens, including vaginal swabs and first-void urine, are crucial for maintaining DNA quality and methylation signal stability. Self-sampling offers a promising alternative to clinician-collected samples, increasing screening coverage and accessibility, especially in underserved populations. However, variability in sample collection devices, storage media, temperature control, and transport duration can lead to DNA degradation and methylation signal attenuation. Standardization of these pre-analytical variables is essential to ensure reproducibility and accuracy of methylation-based assays. For example, studies evaluating methylation markers such as ASCL1/LHX8 in first-void urine have emphasized the need for validated protocols that preserve DNA methylation patterns during sample handling to achieve reliable CIN3+ detection rates comparable to cervical samples (64).

In addition to chemical and procedural optimizations, the choice of sample type and collection method influences DNA quality. Clinician-collected cervical scrapes typically yield higher DNA quantity and quality than self-collected samples, but self-sampling is gaining traction due to its acceptability and potential to improve screening participation. Research has shown that methylation assays can be effectively applied to self-collected specimens when appropriate preservation and processing protocols are employed, although sensitivity may be somewhat lower than that achieved with clinician-collected samples. Therefore, balancing the benefits of increased screening uptake through self-sampling with the technical challenges of maintaining DNA quality is a key consideration in implementing methylation-based screening strategies (65).

Furthermore, the heterogeneity of cervical samples, including the presence of inflammatory cells, mucus, and varying proportions of epithelial cells, can affect DNA extraction efficiency and methylation assay performance. Methods that selectively enrich for epithelial DNA or employ robust normalization controls are necessary to account for sample variability. The use of internal reference genes, such as ACTB, in quantitative methylation-specific PCR assays helps monitor DNA quality and bisulfite conversion efficiency, ensuring reliable interpretation of methylation results (63).

In summary, the integrity and quality of DNA extracted from cervical samples are paramount for the accurate detection of methylation biomarkers in cervical cancer screening. Bisulfite treatment-induced DNA degradation poses a significant challenge, particularly for low-abundance samples. Advances in optimized extraction and conversion protocols, incorporation of protective reagents, and standardization of self-sampling preservation and transport conditions have collectively improved sample utilization and assay sensitivity. Continued refinement of these pre-analytical processes will enhance the clinical applicability of DNA methylation testing, enabling more sensitive, specific, and accessible cervical cancer screening modalities.

### 7.3 Cost and accessibility

The cost and accessibility of gene methylation detection in cervical cancer screening represent critical factors influencing its implementation and widespread adoption, particularly when compared with traditional cytology and HPV testing. Current evidence indicates that methylation detection assays generally incur higher costs than conventional cytological methods but remain less expensive than high-risk HPV (hrHPV) testing. For instance, quantitative methylation-specific PCR (qMSP) assays targeting genes such as PAX1, SOX1, ZNF582, and others have demonstrated promising diagnostic performance with high sensitivity and specificity; however, their operational costs, including reagents, equipment, and technical expertise, contribute to a higher per-test expense relative to Pap smears (66, 67). However, methylation assays offer advantages in terms of automation potential, objective readouts, and reduced reliance on highly trained cytopathologists, which may offset initial costs through improved throughput and consistency.

Cost reduction strategies are essential to enhance the feasibility of methylation testing, especially in low- and middle-income countries (LMICs) where cervical cancer burden is highest. Technological optimization, such as multiplexing multiple gene targets into a single assay, can improve cost-effectiveness by maximizing information yield per sample. For example, multigene panels including FAM19A4, EPB41L3, and PAX1 have shown high diagnostic accuracy and could be adapted for scalable, high-throughput platforms (67, 68). Additionally, the development of simplified PCR-based methods, including methylation-specific PCR (MSP) and quantitative MSP, can reduce reagent consumption and processing time, further lowering costs.

In resource-limited settings, prioritizing single-gene methylation assays or low-cost PCR methods may be a pragmatic approach to gradually introduce methylation testing. For example, assays focusing on PAX1 methylation alone have demonstrated high specificity and sensitivity for detecting high-grade cervical lesions and invasive cancer, suggesting that single-gene tests could serve as an initial triage tool before transitioning to more comprehensive multigene panels as infrastructure and funding improve (69, 10). This stepwise implementation allows for balancing cost constraints with clinical utility, enabling incremental improvements in screening accuracy without overwhelming healthcare budgets.

Governmental policy support and integration into national health insurance schemes are pivotal to expanding access to methylation-based screening. Reimbursement policies that recognize the clinical value of methylation testing can incentivize laboratories and healthcare providers to adopt these assays. For instance, in countries with established cervical cancer screening programs, incorporating methylation testing as a triage method for hrHPV-positive women could be supported by health authorities to reduce unnecessary colposcopy referrals and associated costs (54, 70). Moreover, public health initiatives aimed at educating clinicians and patients about the benefits of methylation testing can facilitate acceptance and demand, further driving economies of scale that reduce per-test costs.

Accessibility is also influenced by the availability of laboratory infrastructure and trained personnel. While methylation assays require molecular biology expertise and equipment such as real-time PCR machines, these technologies are increasingly available even in middle-income countries. The use of self-collected cervical samples for methylation testing has been explored and could enhance accessibility by reducing the need for clinic-based sampling and enabling community-level screening programs (4). Such approaches could be particularly beneficial in rural or underserved areas where access to healthcare facilities is limited.

In summary, although gene methylation detection currently entails higher costs than traditional cytology, it is generally less expensive than HPV testing and offers superior specificity and triage capabilities. Cost reduction through technological optimization, scaling up multiplex assays, and prioritization of single-gene tests in resource-limited settings can enhance affordability. Crucially, government policy support and inclusion in health insurance coverage are key to promoting widespread adoption. Together, these factors can improve the cost-effectiveness and accessibility of methylation-based cervical cancer screening, ultimately contributing to better early detection and reduced cervical cancer morbidity and mortality worldwide.

### 7.4 Methodological heterogeneity and bias risk assessment

Sensitivity and specificity of various methylation biomarkers reported in the current literature for detecting CIN2+ and CIN3+ show significant variability (e.g., 70%–90% sensitivity and 85%–95% specificity). This discrepancy arises not only from tumor heterogeneity but, more importantly, from methodological heterogeneity, specifically manifested in the following: 1. Batch-to-batch differences in bisulfite conversion efficiency, which directly impact downstream PCR amplification efficiency; 2. Inconsistent criteria for determining methylation cutoff values across different studies, leading to varied standards for positive readings; and 3. More critically, most high-sensitivity data currently available originate from retrospective case-control studies. Such studies often suffer from severe selection bias, typically enrolling patients with established diagnoses and known negative controls. Consequently, the diagnostic performance (AUC > 0.9) obtained from these retrospective studies cannot be directly equated to their performance in real-world prospective screening cohorts. Sensitivity estimates lacking prospective validation may be overestimated, a point that requires readers’ attention to avoid undue optimism.

## 8 Future research directions in gene methylation detection

### 8.1 Discovery and validation of novel biomarkers

The identification and validation of novel methylation biomarkers for cervical cancer screening have advanced significantly through the integration of genome-wide methylation sequencing and machine learning algorithms. Genome-wide methylation profiling studies, such as those utilizing the Illumina HumanMethylation450 Array, have revealed distinct methylation signatures that differentiate normal cervical epithelium from cervical carcinoma and high-grade precancerous lesions (CIN3) (30). These studies identified gene panels with hypermethylated CpG sites that serve as robust classifiers for disease progression, underscoring the potential of methylation markers as diagnostic tools. Machine learning approaches, including partial least squares (PLS) modeling, have been instrumental in selecting optimal combinations of methylation markers that enhance sensitivity and specificity in detecting cervical intraepithelial neoplasia and invasive cancer. For example, the GynTect^®^ methylation panel, which includes markers such as ASTN1, DLX1, ITGA4, RXFP3, SOX17, and ZNF671, demonstrated high diagnostic accuracy for CIN3+ lesions, with ZNF671 methylation alone showing an area under the curve (AUC) of 0.811, comparable to the full panel (29). This highlights the feasibility of streamlined methylation assays for clinical triage.

Beyond protein-coding genes, the exploration of non-coding RNAs, particularly long non-coding RNAs (lncRNAs) and circular RNAs (circRNAs), has expanded the repertoire of potential methylation biomarkers in cervical cancer. LncRNAs have been implicated in the pathogenesis of HPV-negative cervical cancers, which tend to have distinct biological behaviors and a poorer prognosis than HPV-positive tumors (71). Aberrant methylation of lncRNA promoters can modulate their expression, influencing tumor progression and serving as candidate biomarkers. Similarly, circRNAs such as circAMOTL1 have been shown to promote cervical carcinogenesis by sponging microRNAs and regulating downstream oncogenic pathways, while their expression may be regulated by epigenetic modifications (72). Although direct evidence of circRNA methylation in cervical cancer remains limited, these findings suggest that integrating non-coding RNA methylation profiles could broaden biomarker panels and improve detection accuracy.

The integration of multi-omics data, combining methylome, transcriptome, and proteome analyses, offers a promising strategy to enhance the precision of cervical cancer screening. Studies have demonstrated that DNA methylation changes correlate with gene expression alterations and protein abundance, reflecting the complex regulatory networks driving carcinogenesis (73). For instance, combining methylation markers with transcriptomic signatures of immune infiltration or oncogenic pathways can refine risk stratification. The cGAS-STING pathway exemplifies this approach, with differential methylation and expression patterns of cGAS and STING genes in cervical tumors being associated with immune cell infiltration and patient prognosis (74). Moreover, proteomic analyses of tumor microenvironment components, such as extracellular matrix scaffolds derived from cervical carcinoma tissues, have revealed molecular features that influence tumor behavior and therapy resistance (75). Integrative models leveraging machine learning can synthesize these diverse data types to construct predictive algorithms with superior sensitivity and specificity compared to single-omic markers.

In summary, the discovery and validation of novel methylation biomarkers for cervical cancer screening have progressed through genome-wide methylation profiling combined with advanced computational methods. Expanding the biomarker landscape to include methylation alterations in non-coding RNAs and integrating multi-omics data hold great promise for developing highly accurate, non-invasive screening tools. These advances are critical for improving early detection, particularly in HPV-negative cases and populations with limited access to conventional screening, ultimately contributing to better clinical outcomes in cervical cancer management.

### 8.2 Liquid biopsy and early detection

Liquid biopsy, particularly the detection of circulating tumor DNA (ctDNA) methylation, has emerged as a promising non-invasive approach for cervical cancer (CC) screening and early detection. ctDNA methylation analysis enables the identification of tumor-specific epigenetic alterations in peripheral blood, offering a minimally invasive method suitable for early-stage cervical cancer and monitoring of recurrence. This approach is especially valuable in low- and middle-income countries (LMICs), where traditional screening methods may be limited by infrastructure and accessibility. For instance, a study conducted among HIV-positive Nigerian women demonstrated that DNA methylation biomarkers identified through epigenome-wide association studies could predict cervical intraepithelial neoplasia (CIN) and CC risk with high sensitivity (92.9%) and specificity (88.6%), underscoring the potential of methylation-based liquid biopsy in high-risk populations (36). The detection of methylated DNA in cervical exfoliated cells also reflects overall cervical lesion status, showing strong concordance with histopathological findings from tissue biopsies. This concordance supports the clinical utility of methylation assays as surrogate markers for cervical disease severity, enabling more accurate risk stratification and management decisions. For example, methylation levels of genes such as PAX1, ZNF671, and others have been shown to increase progressively with lesion severity, correlating well with CIN grades and invasive cancer (52, 60). Advances in liquid biopsy technologies, including digital PCR and next-generation sequencing (NGS), have significantly enhanced the sensitivity and specificity of methylation detection. Digital PCR allows for precise quantification of methylated ctDNA fragments even at low abundance, facilitating early detection of neoplastic changes. NGS-based targeted bisulfite sequencing enables multiplexed analysis of multiple methylation markers simultaneously, improving diagnostic accuracy and enabling comprehensive methylation profiling (60, 76). These technological improvements are driving the evolution of methylation-based screening toward earlier, more convenient, and less invasive modalities. Moreover, methylation assays can be integrated with HPV genotyping to improve triage strategies for hrHPV-positive women, reducing unnecessary colposcopies and overtreatment. For instance, combining methylation markers such as ASCL1/LHX8 or FAM19A4/miR124-2 with HPV16/18 genotyping has demonstrated enhanced sensitivity and specificity for detecting CIN3+ lesions (64). Additionally, methylation testing on self-collected samples, including first-void urine, has shown promising results, expanding the reach of cervical cancer screening to populations with limited access to clinical settings (64). Importantly, methylation markers also hold prognostic value for post-treatment surveillance, as persistent methylation positivity post-therapy correlates with higher risk of recurrence, enabling personalized follow-up strategies (22). In summary, liquid biopsy approaches leveraging ctDNA and cervical exfoliated cell methylation profiling represent a transformative advance in cervical cancer screening and early detection. The high concordance with tissue pathology, combined with the non-invasive nature and technological advancements in detection methods, position methylation-based liquid biopsy as a key tool for improving early diagnosis, risk stratification, and monitoring of cervical neoplasia, particularly in resource-limited settings and high-risk populations.

### 8.3 Artificial intelligence and automated analysis

Artificial intelligence (AI) and automation have emerged as transformative tools in gene methylation detection for cervical cancer screening, offering significant improvements in accuracy, efficiency, and personalized risk assessment. Machine learning algorithms, a subset of AI, have demonstrated the ability to automatically identify complex methylation patterns from high-dimensional datasets, thereby optimizing threshold settings and interpretation of results. For instance, in a study involving DNA methylation biomarkers for cervical intraepithelial neoplasia (CIN) and cervical cancer, machine learning-based classifiers achieved high sensitivity and specificity, such as a DNA methylation classifier that reached 92.9% sensitivity and 88.6% specificity in predicting cervical cancer risk among HIV-positive Nigerian women (36). These algorithms can learn from large training datasets to distinguish subtle methylation differences associated with disease progression, reducing human error and interobserver variability inherent in traditional cytology or histopathology.

Beyond algorithmic analysis, integrated detection platforms combining microfluidic chips with AI analytics have been developed to enable fully automated workflows from sample input to result output. Such platforms streamline the processing of cervical samples, performing DNA extraction, bisulfite conversion, methylation-specific amplification, and data analysis in a seamless, high-throughput manner. This integration not only accelerates turnaround times but also minimizes contamination risks and operator dependency, which is critical for scalability in low-resource settings. For example, recent advances in microfluidics and AI have facilitated the development of point-of-care devices capable of detecting methylation with minimal manual intervention, although specific applications in cervical cancer screening remain under active research.

The power of AI is further amplified when combined with big data analytics, which integrate methylation profiles with clinical and demographic information to build individualized risk prediction models. Such models can stratify patients based on their likelihood of progression from precancerous lesions to invasive cervical cancer, enabling tailored screening intervals and management strategies. For instance, multivariate logistic regression models incorporating methylation markers such as ASCL1/LHX8 in urine samples have shown promising sensitivity and specificity for detecting CIN3+ lesions, supporting their use in personalized screening approaches (64). Moreover, large-scale epigenome-wide association studies (EWAS) have identified novel methylation markers such as PAX1 and NREP-AS1, which, when combined with clinical data, yield high diagnostic accuracy (AUC up to 0.92) for CIN3 and cervical cancer (77). These integrative analyses benefit from AI’s ability to handle heterogeneous data types and uncover complex interactions that may be missed by conventional statistical methods.

Notably, several countries have already incorporated AI-based tools into cervical cancer screening programs. For instance, China has launched pilot projects in multiple provinces using AI-assisted cytology to improve screening efficiency and reduce interobserver variability (78). In the United States, the FDA has cleared AI-driven image analysis systems for cytology triage (79). At the international level, the World Health Organization (WHO) has issued guidelines on digital health interventions, encouraging the use of AI for screening and risk assessment in low-resource settings (80). These examples demonstrate the growing acceptance of AI in clinical practice and provide a basis for integrating methylation data with AI for comprehensive risk prediction.

AI-driven automated analysis also addresses challenges related to sample heterogeneity and variability in methylation levels across populations. For example, methylation markers such as ZNF671 and panels such as GynTect^®^ have been evaluated for their triage performance in hrHPV-positive women, with AI algorithms facilitating the interpretation of methylation signals to improve specificity and sensitivity compared with those of cytology or HPV genotyping alone (29). Additionally, AI models can adjust for confounding factors such as age, HPV genotype, and HIV status, which influence methylation patterns and disease risk, thereby enhancing the robustness of screening tests across diverse cohorts.

Furthermore, AI applications extend to image-based diagnostics related to cervical neoplasia, complementing methylation analyses. Deep learning models have been successfully applied to digital histopathology images to grade cervical intraepithelial neoplasia with high accuracy (up to 94.57%), reducing interobserver variability and enabling rapid, standardized assessments (81). When combined with methylation data, these multimodal AI approaches hold promise for comprehensive, non-invasive cervical cancer screening and diagnosis.

In summary, the integration of AI and automation in gene methylation detection for cervical cancer screening represents a paradigm shift toward more precise, efficient, and personalized healthcare. Machine learning algorithms enhance the detection and interpretation of methylation patterns, while integrated platforms enable fully automated workflows suitable for large-scale screening. Big data analytics facilitate the development of individualized risk models by combining molecular and clinical data. Together, these advances promise to improve early detection, reduce unnecessary interventions, and optimize resource allocation, particularly in low-resource settings where cervical cancer burden remains high. Ongoing research and validation studies are essential to translate these technological innovations into routine clinical practice, ensuring accessibility and equity in cervical cancer prevention worldwide.

## 9 Ethical and policy considerations in gene methylation testing

### 9.1 Informed consent and privacy protection in methylation testing

Methylation testing in cervical cancer screening involves the analysis of epigenetic modifications in host-cell DNA, which inherently includes sensitive genetic information. Given the nature of this testing, it is imperative to ensure that patients are fully informed about the purpose of the methylation assay, the clinical significance of the results, and any potential risks associated with the testing process. Patients must understand that methylation markers, such as those identified in genes such as PAX1 and NREP-AS1, serve as biomarkers for detecting cervical intraepithelial neoplasia (CIN) and invasive cervical cancer, offering improved diagnostic accuracy over traditional cytology or HPV testing alone (77). Clear communication about the implications of positive or negative methylation results is essential to empower patients to make informed decisions regarding their health management.

Establishing a robust data security framework is critical to protecting methylation data against unauthorized access, misuse, or breaches. Since methylation profiles can reveal not only cancer risk but also broader genetic and epigenetic information, strict confidentiality protocols must be implemented. This includes secure storage of methylation data, controlled access limited to authorized healthcare professionals, and compliance with relevant data protection regulations. The potential for data misuse, such as discrimination or stigmatization based on genetic information, necessitates proactive measures to prevent such outcomes. For example, integrating methylation testing within established cervical screening programs requires adherence to privacy standards that protect patient identity and the integrity of genetic data (65).

When methylation testing yields positive results indicating high-grade lesions or cancer risk, it is crucial to provide patients with professional counseling and psychological support. The detection of methylation changes may provoke anxiety or distress, especially given the complex nature of epigenetic information and its prognostic implications. Healthcare providers should be trained to deliver results sensitively, explaining the meaning of methylation positivity in the context of cervical cancer risk and the subsequent steps for diagnosis and treatment. Psychological support services should be accessible to help patients cope with the emotional impact, reducing the likelihood of excessive anxiety or misunderstanding. This approach aligns with patient-centered care principles and enhances adherence to follow-up procedures, ultimately improving clinical outcomes (82).

Moreover, the integration of methylation testing into cervical cancer screening programs must consider the diverse patient populations, including transgender and gender-diverse individuals who retain a cervix and are at risk for cervical cancer but may face barriers to screening participation (83). Ensuring informed consent processes are inclusive and culturally sensitive is vital to address the unique concerns of these groups. Providing clear information about methylation testing and its role in screening can help overcome mistrust or apprehension, fostering equitable access to preventive care.

In resource-limited settings, where cervical cancer burden is disproportionately high, the implementation of methylation testing raises additional ethical considerations. While methylation markers offer promising triage tools with high sensitivity and specificity, the infrastructure for secure data management and patient counseling may be limited (84). Strategies to protect patient privacy must be adapted to local contexts, balancing technological capabilities with ethical obligations. Training healthcare workers in informed consent and confidentiality practices is essential to maintain trust and ensure ethical standards are upheld. Please refer to **Table 1** for details.

**Table 1:** Comparative analysis of methylation testing and HPV genotyping in clinical implementation.

Clinical implementation	*Methylation testing*	*HPV genotyping*
*Structure requirements*	Professional nucleic acid extraction and bisulfite conversion equipment/automated platforms are required	Conventional PCR laboratory
*Throughput*	Currently, it is still mainly single-tube/small-scale, with a high dependence on high-throughput automation	High-throughput, highly automated
*Single-test cost*	Lower	Higher
*Impact on clinical outcomes*	The risk of over-treatment is high	Optimize precise traffic diversion

Finally, the evolving landscape of cervical cancer screening, including the use of self-sampling and molecular testing, underscores the need for ongoing patient education about new technologies such as methylation assays (85). Transparent communication about the benefits and limitations of methylation testing, alongside traditional screening methods, supports informed decision-making and respects patient autonomy. As methylation testing becomes more integrated into clinical practice, continuous evaluation of consent procedures and privacy protections will be necessary to address emerging challenges and maintain ethical integrity in cervical cancer prevention.

### 9.2 Screening guidelines and policy development

The integration of DNA methylation detection into cervical cancer (CC) screening guidelines and policy frameworks represents a critical advancement in improving early diagnosis and management of this malignancy. International organizations such as the World Health Organization (WHO) and the International Federation of Gynecology and Obstetrics (FIGO) are pivotal in setting global standards for CC screening. Current evidence strongly supports the need for these bodies to update their screening guidelines to incorporate methylation-based assays as recommended triage tools alongside or after primary human papillomavirus (HPV) testing. For instance, methylation panels targeting genes such as PAX1, SOX1, JAM3, and ZNF671 have demonstrated high diagnostic accuracy for cervical intraepithelial neoplasia grade 2 or worse (CIN2+), with area under the curve (AUC) values often exceeding 0.85, and sensitivities and specificities surpassing those of traditional cytology in various cohorts (86–88). These findings underscore the potential of methylation assays to refine risk stratification, reduce unnecessary colposcopy referrals, and improve patient outcomes. Consequently, WHO and FIGO guidelines should formally endorse methylation testing as a triage strategy, especially for HPV-positive women with low-grade or negative cytology, to optimize clinical decision-making and resource allocation.

At the national level, countries must tailor cervical cancer screening policies to their specific healthcare infrastructure, economic capacity, and population risk profiles. Stratified screening strategies that combine HPV primary screening with methylation-based triage offer a pragmatic approach to balancing sensitivity, specificity, and cost-effectiveness. For example, a tiered algorithm employing HPV testing as the initial screen followed by methylation marker assessment for HPV-positive women can effectively identify those at highest risk for CIN2+ lesions, thereby prioritizing them for colposcopy and treatment (87, 89). This approach is particularly advantageous in low- and middle-income countries (LMICs), where cytology-based screening programs face challenges such as limited laboratory infrastructure and trained personnel. Moreover, methylation testing can be applied to self-collected samples, which has been shown to improve screening uptake and reduce loss to follow-up in LMICs, further supporting its inclusion in national screening policies (90). Countries with varying resource levels should thus develop flexible, evidence-based screening algorithms that incorporate methylation assays to maximize coverage and effectiveness. Please refer to **Table 2** for details.

**Table 2:** Summary of common methylation biomarkers and their clinical translation pathways.

Methylation biomarker	Applicable detection technology platform	Function	Clinical implementation maturity and practical recommendations
PAX1/SOX1	qMSP	Triage of hrHPV-positive women to identify those requiring immediate colposcopy.	High maturity. Validated in multiple retrospective and prospective cohorts. Exhibits excellent specificity (~90%), reducing unnecessary referrals. Cost-effective and highly suitable for routine clinical labs and resource-limited settings.
FAM19A4/miR124-2	Multiplex qMSP	Reflex triage in primary HPV-based screening; also applicable to post-treatment surveillance.	High maturity. Validated in large European multicenter trials (e.g., POBASCAM). Demonstrates excellent PPV (50%–70%) and reduces colposcopy referrals by up to 50%–70%. Suitable for fully automated, high-throughput screening platforms.
EPB41L3 + HPV L1/L2	Targeted bisulfite sequencing/multiplex qMSP	Primary screening and long-term risk stratification (predicting incident CIN3+ up to 5 years before diagnosis).	Moderate-to-high maturity. Provides robust predictive power for high-grade lesions. However, it requires sophisticated NGS or multiplex equipment, making it more viable in tertiary care centers or well-equipped regional laboratories.
Whole-genome methylation map	NGS		Exploratory research. Not suitable for initial screening; can be used as a tool for scientific research and discovery of novel biomarkers

To facilitate the clinical translation of methylation-based screening, regulatory and reimbursement frameworks must evolve accordingly. Accelerating the registration and approval of methylation test kits is essential to ensure their availability and standardization across healthcare settings. Regulatory agencies should establish clear criteria for analytical and clinical validation of methylation assays, drawing on robust clinical trial data demonstrating their diagnostic performance and clinical utility (68, 88). Parallel efforts are needed to integrate these tests into health insurance reimbursement schemes, reducing financial barriers for patients and healthcare providers. Inclusion of methylation testing in national essential diagnostics lists and coverage by public health insurance would incentivize adoption and scale-up. Additionally, policy initiatives should promote both the training of healthcare workers in the interpretation and application of methylation test results and public education campaigns to raise awareness of the benefits of advanced molecular screening methods. Collectively, these measures will accelerate the incorporation of methylation detection into routine cervical cancer screening, ultimately contributing to earlier diagnosis, improved patient outcomes, and progress toward global cervical cancer elimination goals.

## 10 Conclusion

The evolution of gene methylation detection as a novel tool for cervical cancer screening represents a significant advancement in the field of oncology diagnostics. From an expert perspective, this technology addresses critical limitations inherent in traditional screening methods, such as cytology and HPV DNA testing, by offering enhanced specificity, objectivity, and the potential for automation. These attributes collectively contribute to more accurate risk stratification, which is essential for optimizing patient management and reducing unnecessary interventions.

The integration of host gene methylation markers, including PAX1 and SOX1, with HPV gene methylation profiles has demonstrated considerable clinical utility. These biomarkers enable effective triage of HPV-positive individuals, thereby refining the screening cascade and focusing resources on those at genuine risk of progression to cervical intraepithelial neoplasia or invasive cancer. Moreover, their applicability in self-sampling contexts expands access to screening, particularly in underserved populations, while their role in postoperative surveillance offers a promising avenue for the early detection of residual or recurrent disease. This multifaceted utility underscores the transformative potential of methylation markers in both primary and secondary prevention paradigms.

Technological advancements such as quantitative methylation-specific PCR (qMSP), next-generation sequencing (NGS), and digital PCR have been instrumental in translating methylation detection from research settings into clinical practice. These methodologies provide the sensitivity and quantitative precision necessary for reliable biomarker assessment. However, despite these technical strides, challenges remain. Standardization across laboratories is imperative to ensure reproducibility and comparability of results, which is crucial for widespread clinical adoption. Additionally, cost considerations must be addressed to facilitate equitable access, especially in low-resource settings where cervical cancer burden is disproportionately high. Ethical concerns, including informed consent and data privacy related to genetic and epigenetic information, also warrant careful deliberation to maintain patient trust and comply with regulatory frameworks. We must objectively recognize that although DNA methylation detection has shown great potential in laboratory research, there are still three major gaps between the identified biomarkers and their widespread clinical application in the real world: cost control, equipment accessibility, and standardized quality control. Instead of overemphasizing the absolute advantages of the technology itself, we should address its practical challenges within the existing healthcare system. Future research should focus on conducting large-scale, prospective, multicenter clinical trials to generate clinical efficacy data and on performing cost-effectiveness analyses from a health economics perspective.

Balancing these diverse research perspectives and findings requires a nuanced approach. While the promise of methylation-based screening is compelling, it is essential to integrate these tools within existing healthcare infrastructures thoughtfully. This includes validating novel biomarkers through large-scale, prospective studies and harmonizing assay protocols to establish clinical guidelines. Furthermore, the incorporation of liquid biopsy techniques offers a minimally invasive alternative that could revolutionize screening and monitoring, enhancing patient compliance and enabling dynamic disease tracking. The convergence of artificial intelligence with methylation data analysis presents another frontier, potentially augmenting diagnostic accuracy through sophisticated pattern recognition and risk prediction models.

Future research should prioritize the discovery of new methylation markers with higher sensitivity and specificity, alongside efforts to optimize sample collection and processing methods. The development of multiplexed assays capable of simultaneously evaluating multiple biomarkers could streamline workflows and reduce costs. Importantly, fostering multidisciplinary collaboration among molecular biologists, clinicians, bioinformaticians, and health policy experts will be critical to overcoming existing barriers and accelerating clinical translation.

Policy support plays a pivotal role in this endeavor. Investment in infrastructure, reimbursement frameworks, and public health initiatives can facilitate the integration of gene methylation detection into national cervical cancer screening programs. Educational campaigns aimed at healthcare providers and the public are equally vital to promote acceptance and understanding of these novel technologies. Ultimately, the successful implementation of methylation-based screening has the potential to substantially reduce the global burden of cervical cancer by enabling earlier detection, personalized risk assessment, and tailored interventions.

In conclusion, gene methylation detection embodies a promising frontier in cervical cancer screening, offering a sophisticated, objective, and scalable approach that complements and enhances existing methodologies. The path forward necessitates a balanced synthesis of technological innovation, rigorous clinical validation, ethical oversight, and policy-driven support. By addressing these multifactorial challenges through coordinated efforts, the medical community can harness the full potential of methylation biomarkers to improve patient outcomes and advance global cervical cancer control.
